# Sharpening the Coach’s Eye: An Observational Study Investigating the Trainability of An Eye-Tracking Strategy for Perceiving Barbell Velocity Loss in Resistance Training

**DOI:** 10.1186/s40798-026-01003-2

**Published:** 2026-03-11

**Authors:** Asaf Ben-Ari, Scott Henry, Israel Halperin, Laura Carey, Antonio Dello Iacono

**Affiliations:** 1 Sport and Physical Activity Research Institute (SPARI), Division of Sport, Exercise and Health, School of Health and Life Sciences, University of the West of Scotland, Glasgow, UK; 2 Department of Health Promotion, School of Public Health, Faculty of Medical and Health Sciences, Tel Aviv University, Tel Aviv-Yafo, Israel; 3 Sylvan Adams Sports Institute, Tel Aviv University, Tel Aviv-Yafo, Israel

**Keywords:** Resistance training, Velocity-based training, Eye-tracking, Gaze strategy training

## Abstract

**Background:**

Resistance training (RT) coaches can implement velocity-based training (VBT) to prescribe set volume by terminating sets at a target velocity loss (VL) threshold. However, VBT necessitates velocity-tracking devices that demand time and expertise. In a previous study, we asked RT coaches to detect VL thresholds using solely their observational judgment – the Coach’s Eye. We found improved accuracy when participants spontaneously used a “bar strategy” (focusing on the barbell). Here, we investigated whether coaches could be trained to intentionally use this strategy and whether it would be associated with greater accuracy in detecting VL thresholds.

**Methods:**

Twenty RT coaches completed one experimental session involving a gaze strategy training intervention. Participants watched an instructional video on the bar strategy, practiced with gaze feedback, and completed a VL detection task. The task involved watching videos of trainees performing bench presses and back squats and detecting 20% and 40% VL thresholds. We examined the frequency of bar strategy use using a one-sided exact binomial test and the accuracy in VL thresholds detection using a negative binomial mixed-effects model.

**Results:**

Participants used the bar strategy in most trials (mean proportion = 78.81, 95% CI [0.75, 1.00]). The average absolute error in detecting VL thresholds was 1.5 (SD = 2.3) repetitions. Error decreased when using the bar strategy (-1.23, 95% CI [-1.99, -0.03]), detecting 40% VL thresholds (-1.60, 95% CI [-1.88, -1.26]) compared to 20%, and observing sets loaded with 85% 1RM (-1.59, 95% CI [-1.93, -1.16]) but not with 65% 1RM (-0.48, 95% CI [-1.01, 0.18]) compared to 45% 1RM. Lastly, mental fatigue did not significantly affect accuracy (-0.01, 95% CI [-0.03, 0.01]).

**Conclusions:**

This study provides novel evidence that, under laboratory conditions, RT coaches can be trained to use a gaze strategy associated with improved accuracy in detecting barbell VL. Thus, the Coach’s Eye may offer a practical, coach-led complement to velocity-tracking devices in VBT, with further studies in real-world settings required to establish its ecological validity.

**Supplementary Information:**

The online version contains supplementary material available at 10.1186/s40798-026-01003-2.

## Key Points

We investigated the trainability of a gaze strategy for tracking barbell movement velocity loss (VL) during resistance training (RT) and its association with accuracy in detecting specific VL thresholds.

A short instructional video, followed by practice with gaze feedback, successfully trained RT coaches to use the gaze strategy, which was associated with greater accuracy in detecting VL thresholds.

The study’s findings suggest that RT coaches can be trained to accurately use their observational judgment to detect VL thresholds with the potential to serve as an alternative to velocity-tracking devices.

**Supplementary Information:**

The online version contains supplementary material available at 10.1186/s40798-026-01003-2.

## Background

Velocity-based training (VBT) is a resistance training method (RT) that utilizes movement velocity to adjust training variables in real-time according to the athlete’s actual performance [1]. One application of VBT is the use of velocity loss (VL) to determine set volume (i.e., the number of repetitions in a set) [1, 2]. When using VL to adjust set volume, RT coaches can prescribe trainees to perform repetitions until their velocity drops below a percentage threshold (e.g., 20%) anchored to the first repetition in the set (i.e., 100%) [3]. This application of VBT operates on the principle that VL is a proxy for neuromuscular fatigue, with an inverse linear relationship between the number of repetitions performed and the associated velocity outputs [4]. In other words, as fatigue accumulates with each additional repetition, movement velocity declines. Therefore, when using VL to determine set volume, training is adjusted in real-time to match each trainee’s capability, ensuring a relatively consistent exertion within and between sessions [5]. This individualized approach may enhance training effectiveness and improve performance outcomes [6–8]. However, implementing VL and VBT presents several barriers, as the required velocity-tracking devices can be technically demanding and time-consuming, potentially disrupting the coaching process, especially in group settings [9].

To address these barriers and develop a VBT approach that is independent of technology, several studies have explored using trainees’ perceived VL as an alternative to direct measurements [10–17]. Some studies focused on developing velocity perception scales to help trainees estimate the exact velocity of a repetition (e.g., 1 m⋅s^− 1^) [10–13]. Others examined trainees’ ability to estimate the precise VL percentage of a repetition, relative to the first repetition in a set (e.g., 10% VL) [14], and to detect the repetition at which velocity drops below a predefined threshold (e.g., 20% VL threshold) [15–18]. For example, Dello Iacono et al. asked trainees to detect the repetitions exceeding 20% and 40% VL thresholds relative to the first repetition in a set during a bench-press exercise [17]. Comparisons between trainees’ perceived and actual VL measurements revealed an average absolute error range of 1–2 repetitions [17]. Similarly, Shaw et al. found that most trainees underestimated a 20% VL threshold during deadlifts at 60% and 80% of one-repetition maximum (1RM), while those who overestimated the threshold had an average error of 0–1.4.4 repetitions [16]. Overall, these error ranges suggest that trainees’ perceptions of VL thresholds are relatively accurate. Yet, even such small errors can meaningfully influence training outcomes depending on the set volume used, with a greater impact in shorter sets and a lesser impact in longer ones. Accordingly, the use of perceived VL for prescribing and monitoring set volume in RT should be considered on a case-specific basis.

While these studies highlight the potential of perceived VL as a practical, tech-free alternative to traditional VBT, they have primarily focused on trainees, overlooking RT coaches’ ability to perceive VL. In many real-world RT scenarios, it is the coach, not the trainee, who decides when to end a set, often based on visual cues such as changes in technique, signs of fatigue and exertion, and proximity to task failure. Therefore, to expand the applicability of the perceived VL approach, we recently investigated whether RT coaches could accurately detect repetitions corresponding to predefined VL thresholds using only their observational judgment – “the "Coache's Eye” [19]. In controlled laboratory settings, 20 certified RT coaches watched video recordings of trainees performing RT exercises. We asked them to detect the repetitions corresponding to VL thresholds of 20% and 40% relative to the first repetition in a set. Eye-tracking glasses were used to record the coaches’ gaze during the task, allowing for the exploration of effective gaze strategies. Results showed an absolute average error of 2.6 repetitions. Notably, eye-tracking analysis revealed that in about 48% of observations, coaches used a “bar strategy” – focusing their gaze on the barbell or the attached weight plates – and demonstrated 1.7 repetitions greater accuracy compared to when they focused elsewhere (“no-bar strategy”). Moreover, the absence of specific guidance regarding gaze direction prompts the question of whether providing explicit instructions to focus on the barbell could further enhance accuracy.

Therefore, in the current study, we aimed to extend our previous findings by investigating (1) whether RT coaches can learn and intentionally implement the bar strategy, and (2) whether doing so is associated with greater accuracy in detecting VL thresholds. To explore these questions, we recruited certified RT coaches to participate in a single experimental session. During this session, we introduced them to the bar strategy through an instructional video, provided practice opportunities with gaze feedback, and had them complete a VL thresholds detection task. We hypothesised that following this gaze training intervention, most coaches would successfully adopt the bar strategy and, based on our previous findings, that doing so would be associated with greater accuracy in detecting VL thresholds.

## Methods

### Sample Size and Participants

Based on our previous study, we aimed for a convenience sample size of 20 participants. To ensure this sample size would provide adequate statistical power, we conducted a simulation-based power analysis [20]. This approach involved three steps: (1) simulating new datasets, (2) analyzing each dataset to test for statistical significance, and (3) calculating the proportion of simulations that yielded significant results. To stimulate the new data sets, we used the estimates from the model in our previous study [19]. Specifically, we assessed the statistical power for detecting the observed effect size of our primary outcome of interest: Coach’s Eye accuracy, as a function of eye-tracking strategy (bar vs. no-bar). Based on 1000 simulations, each consisting of 960 observations (20 participants × 24 videos × 2 VL thresholds per video) and an alpha level of 0.05, the study’s statistical power was estimated to be 100.0% (95% CI 99.63, 100.0).

Accordingly, we recruited 20 certified RT coaches to participate in the study (17 males; 10 with VBT experience; mean age 29.3 ± 7.2). Since the participants in the current study also participated in our previous study, to mitigate any learning effects or retention bias, we ensured a minimum washout period of eight weeks between studies. All participants provided written informed consent after receiving an oral explanation of the study’s purpose and potential risks. All procedures were approved by the Ethics Committee of the University of the West of Scotland (approval number: 2024–16384).

### Procedures

Participants attended the laboratory for a single experimental session. The session began with a 9-minute, step-by-step instructional video that trained participants on how to implement the bar strategy for detecting VL. This video, as well as all the other videos of the experimental session, was projected onto a large screen (4 × 1.5 m) in a quiet laboratory environment.

**Fig. 1:**
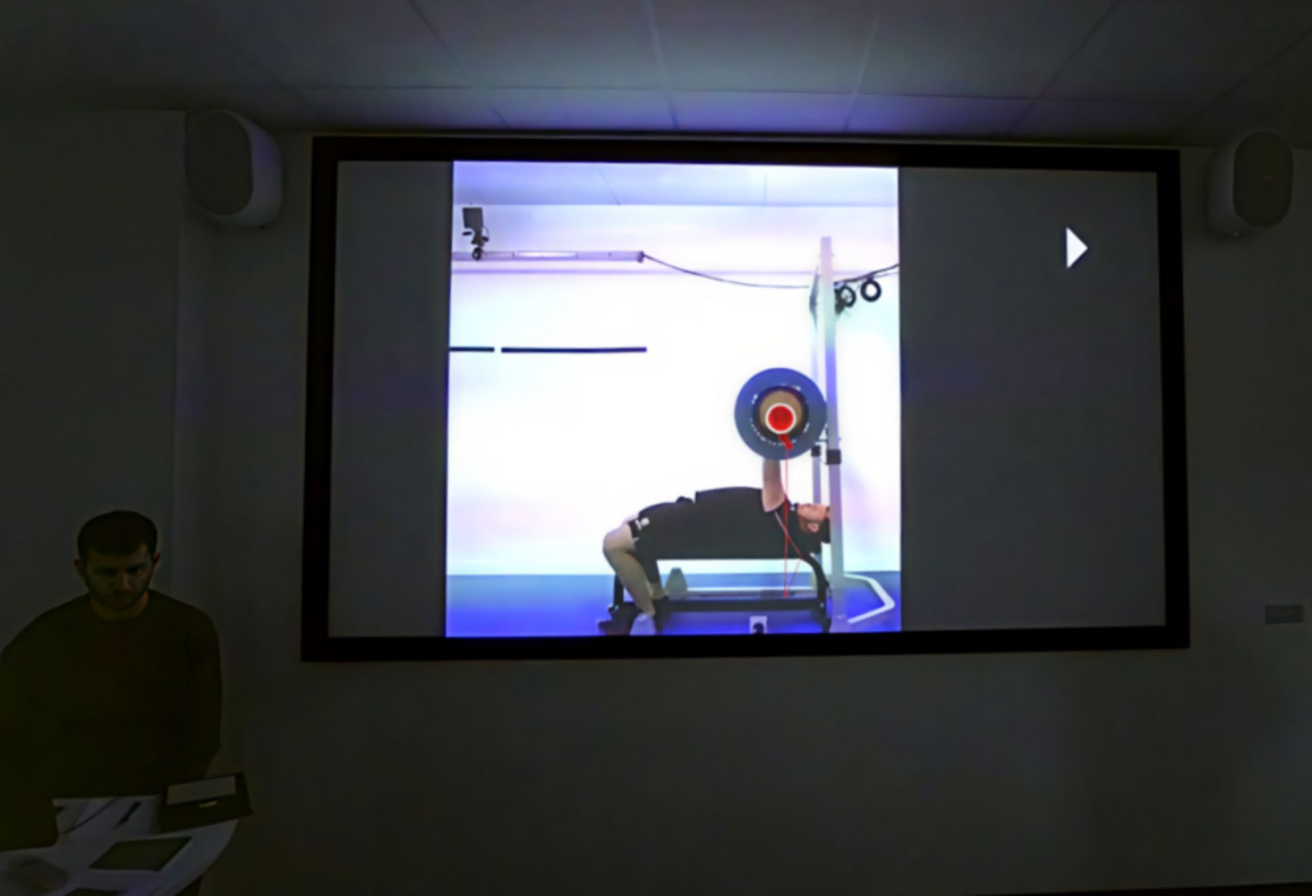
A screenshot of the eye-tracking glasses’ proprietary app (Tobii Pro Glasses 3 Controller App, version 1.19.4) showing the glasses’ recordings. The red circle highlights the participant’s gaze focus, providing a visual representation of where the person was looking on the screen

After watching the video, a researcher assisted participants in putting on eye-tracking glasses (Tobii Glasses 3, Tobii Technology, Danderyd, Sweden) and calibrated the devices. Then, participants practiced the bar strategy using two videos featuring a trainee performing the barbell bench press (filmed from a side point of view) and the barbell back squat (filmed from a front point of view). The practice videos featured a different trainee than those in the experimental videos. The participants stood three meters from the screen and were instructed to indicate (by saying “stop”) the repetition at which they perceived a 20% and 40% decrease in the concentric phase’s barbell velocity compared to the first repetition. After each practice video, the researcher provided participants with feedback on their gaze by showing them the recordings from the eye-tracking glasses using the glasses’ proprietary app (Tobii Pro Glasses 3 Controller App, version 1.19.4). The app displayed the glasses’ recordings, with the participant’s gaze focus highlighted by a red circle, providing a visual representation of where the person was looking on the screen (Fig. 1). The researcher reviewed the recordings alongside the participants and verified whether they successfully implemented the bar strategy. This was determined by checking that the red circle (participants’ gaze) was located on the barbell when watching a front-view recording and on the weight plates when watching a side-view recording. The aim of the feedback was solely to practice implementing the bar strategy; therefore, input on perceived VL accuracy was not provided. Once practice was completed, participants took a 5-minute break.

After the 5-minute break, participants watched 24 experimental videos in a randomized order. The videos showed two trainees (one male and one female) performing the barbell bench press and the barbell back squat exercises with three progressively heavier loads (described further in the Research Tools section). The participants stood three meters from the screen and were instructed to indicate (by saying “stop”) the repetition at which they perceived a 20% and 40% decrease in the concentric phase’s barbell velocity compared to the first repetition, while implementing the bar strategy they had just learned. Not all videos included both 20% and 40% VL; some included only a 20% VL, while others included neither. We provided this information to participants before the session began. Immediately after each answer, or at the end of a video, if no answer was given, the researcher paused the video, noted the repetition number, and asked participants to rate their perceived mental fatigue using a visual analog scale.

At the end of the session, we asked participants two questions: (1) “How accurate do you think you were in detecting the VL thresholds?”; (2) “Did you implement the new strategy? If so, how effective were you in implementing it?”. Participants were asked to rate their answers on a scale ranging from 0 (“not accurate”/“not effective”) to 100 (“accurate”/“effective”).

#### Research Tools

**Fig. 2:**
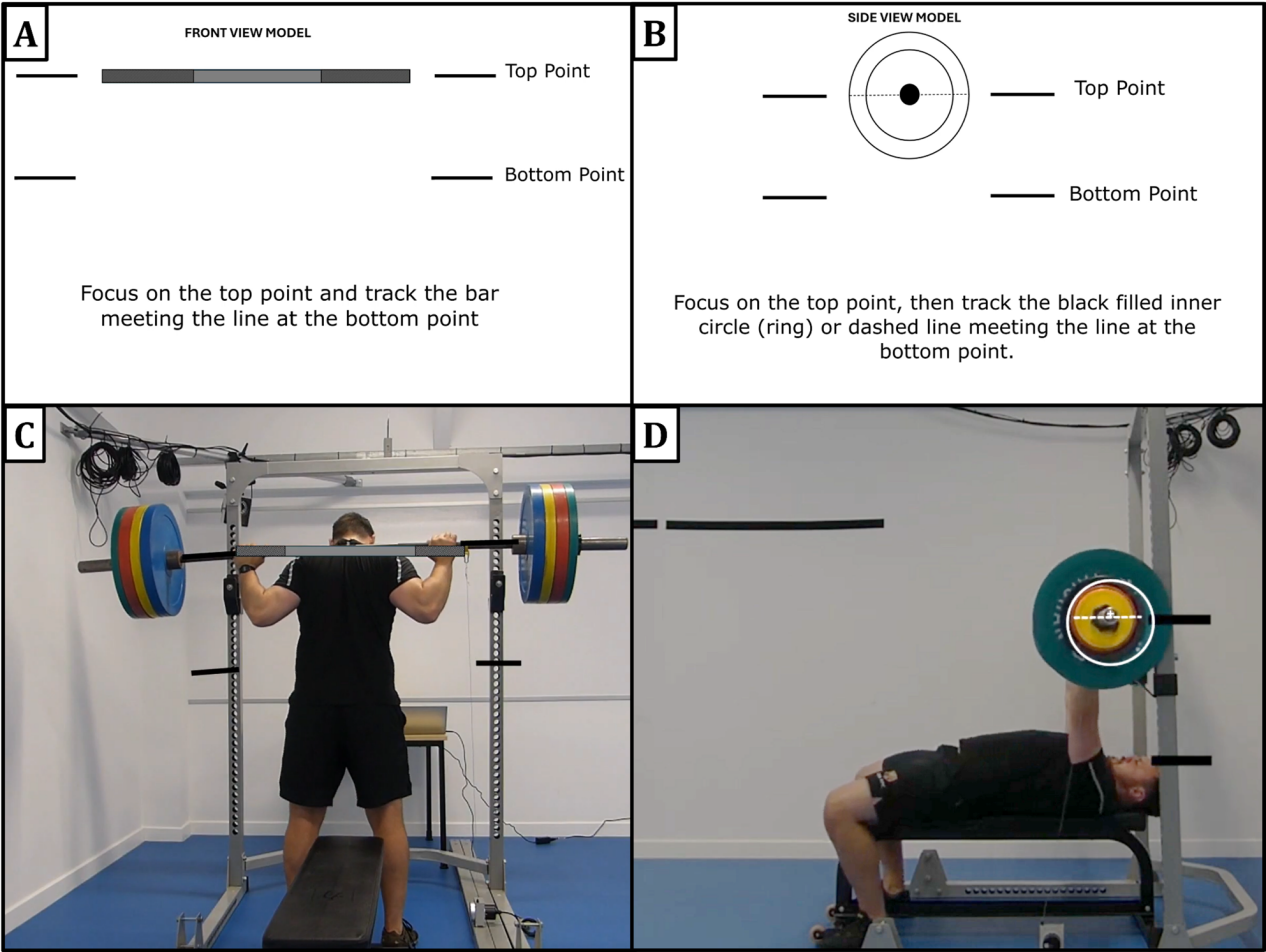
Screenshots from the gaze training instructional video. (**A**) Animated schematic model of velocity tracking technique for the front point of view, and (**B**) side point of view. (**C**) The schematic model overlaid on a real trainee performing the back squat, recorded from the front point of view, and (**D**) performing the bench press, recorded from the side point of view

*Instructional Video*: The video begins by showing a trainee performing back squats (front view) and bench presses (side view), introducing the concept of the “anchor rep” (first repetition in a set) as a 100% reference point for velocity comparison. Next, animated schematic models demonstrate velocity tracking techniques for each point of view: for the front point of view, following a bar-shaped object’s trajectory (Fig. 2A); for the side point of view, following either the center of a plate-shaped object or an imaginary horizontal line across the plate’s diameter (Fig. 2B). The animated models first include visual guides (two lines) representing the lower and upper extremes of the object’s range of movement, and later instruct the observer to imagine them. To calibrate the perception of VL, the models display the object’s movement in decreasing velocity, with labels indicating the repetitions associated with 20% and 40% VL. Finally, the schematic models are overlaid on a real trainee performing the exercises (Fig. 2C–D). At the end of the video, three recap questions are presented on the screen, and read aloud by the researcher, to ensure participants’ understanding: (1) “What is the anchor rep?”, (2) “Which are the range points when tracking the bar?” and (3) “What object should you imagine when tracking the lift?”. The instructional video is available here: https://www.youtube.com/watch?v=RvZC-K8qLXU.

*Experimental Videos*: The video recordings featured two trainees (one female, one male), both with at least 5 years of RT experience and who trained regularly, at least 3 times per week. The trainees performed two exercises: barbell back squat and barbell bench press, with three progressively heavier loads – 45%, 65%, and 85% of their 1RM – and were recorded from both a front and a side point of view. The trainees were instructed to perform the concentric phase of each repetition (i.e., upward movement) as fast as possible and terminate each set when reaching self-selected task failure (the point at which they felt they could not complete an additional repetition). All exercises were performed with a free 20-kg barbell (Origin, Edinburgh, United Kingdom) and calibrated weight plates (Eleiko, Halmstad, Sweden). Concentric barbell velocity was recorded for every repetition using a linear positioning transducer (Chronojump Boscosystem, Barcelona, Spain) attached to the barbell with a string tether positioned perpendicular to the ground. Additional information regarding the recording procedure for the videos and the featured trainees can be found in the Supplementary File.

*Mental Fatigue Scale*: Participants were asked to report their mental fatigue (“How mentally fatigued are you?”) using a visual analog scale ranging from 0 (“no fatigue”) to 100 (“maximal fatigue – I can’t carry on the task”) [21]. An electronic version of the scale (Supplementary File: Figure S1) was displayed on the screen immediately after each answer and/or at the end of the video if participants provided no answer regarding the detection of VL.

*Eye-Tracking:* We used the Tobii Pro Glasses 3 (Tobii Technology, Danderyd, Sweden), lightweight head-mounted glasses, to track participants’ gaze at a sampling rate of 100 Hz. The glasses feature a forward-facing scene camera that records the participant’s field of view at 25 frames per second with a resolution of 1920 × 1080 pixels. A researcher fitted the glasses to each participant and calibrated them using the Tobii Pro Glasses Controller software (firmware version 1.19.1). Calibration was conducted by instructing participants to direct their gaze at a black dot printed on a calibration card positioned 1 m in front of them.

We performed the eye-tracking analysis using Tobii Pro Lab Analyzer software (version 1.24.54542). First, we applied a built-in fixation filter to the raw gaze data, which displayed a red circle on the gaze video to indicate fixations. Next, we manually coded (frame-by-frame) the concentric phase of each lift in the video, recording the fixation duration of each of the following predefined areas of interest: the bar, weight plates, and any other location (e.g., lifter’s body, wall markings, etc.). For analysis, these areas were collapsed into two categories: “bar” (the bar, weight plates, and ring) and “no-bar” (any other location). A gaze was classified as a bar strategy if > 50% of the fixation during the concentric phase was directed at the bar, weight plates, or ring; otherwise, it was classified as a no-bar strategy. The primary analysis was conducted by a single researcher (LC). In most cases, gaze strategy classification was straightforward. When uncertainty arose regarding the classification of a lift, a second researcher (AB) independently reviewed the video recording. If agreement was reached, the classification was finalised accordingly; if disagreement persisted, a third researcher (ADI) reviewed the recording, and the classification was determined by consensus.

Gaze strategy was coded for two specific repetitions in each set (one for each VL threshold), depending on whether and when the VL threshold was reached and reported. If a participant reported a repetition for a VL threshold, regardless of its accuracy, we coded the gaze strategy used during that repetition. For example, if a participant reported a 20% VL at the 8th repetition, we coded their gaze strategy during the 8th repetition, even if the actual VL was reached earlier or later in that set. If a participant did not report any repetition, but the VL threshold was in fact reached, we coded their gaze strategy during the actual repetition at which the threshold was reached. For example, if the 20% VL occurred at the 8th repetition and the participant gave no response, we coded their gaze strategy during the 8th repetition. Finally, when a VL threshold was not reached, and the participant did not report any repetition, we coded their gaze strategy during the last repetition of the set.

##### Statistical Analysis

The first primary outcome measure in this study was the proportion of trials classified as a bar strategy. We treated gaze strategy as a categorical variable (no-bar, bar) and used a one-sided exact binomial test to examine whether this proportion exceeded the chance level of 0.5.

The second primary outcome measure in this experiment was the Coaches’ Eye accuracy, defined as the absolute error between the perceived repetitions (reported by the participant) and the actual repetitions (measured via the linear positioning transducer) at which velocity exceeded the 20% and 40% loss thresholds. For example, if a 20% VL occurred at the 6th repetition and a participant reported it at the 8th, the absolute error would be 2 repetitions. When a participant did not report a perceived VL, accuracy was calculated as the absolute difference from the total number of repetitions completed by the trainee. For example, if a 40% VL occurred at the 12th repetition in a 15-repetition set, and the participant did not indicate any repetition, the absolute error would be 3 repetitions. Lastly, if a participant indicated repetitions exceeding the VL thresholds when no such loss occurred in the set, the error was also calculated as the absolute difference from the total number of repetitions completed by the trainee. For example, if a participant reported a 40% VL occurring at the 8th repetition when it did not occur in a 12-repetition set, the absolute error would be 4 repetitions.

Accuracy was treated as discrete count data, with a lower bound of zero (perfect accuracy) and no theoretical upper bound (indicating lower accuracy). Exploratory data analysis revealed that the data were right-skewed, with a considerable number of zeros, and overdispersed, with variance considerably larger than the mean of the outcome measure. Consequently, we fitted a generalized linear mixed-effects regression model with a log link function, specifying a negative binomial error distribution, to examine the effects of VL threshold, lifted load, gaze strategy, and mental fatigue on the outcome accuracy. Accordingly, the following regression model was fitted:

$$\begin{aligned} \log \left( {accuracy_{{in}} } \right){\text{ }} & = {\text{ }}b_{{0in}} + {\text{ }}b_{{1 - 2}} threshold{\text{ }} + {\text{ }}b_{{3 - 5}} load{\text{ }} \\ & + {\text{ }}b_{{6 - 7}} strategy{\text{ }} + {\text{ }}b_{8} fatigue{\text{ }} + \varepsilon _{{in}} \\ \end{aligned}$$

In this model, accuracy_in_ was the repeated-measures outcome for each participant (continuous count variable); b_0in_ represented a participant-specific random intercept, accounting for repeated measurements and between-subject variability; b_1–2_ represented the fixed effect of VL threshold (categorical variable: 20%, 40%); b_3–5_ represented the fixed effect of lifted load (categorical variable: 45%, 65%, 85% of 1RM); b_6–7_ represented the fixed effect of gaze strategy (categorical variable: no-bar, bar); b_8_ represented the fixed effect of mental fatigue (continuous variable: 0–100), and; ε_i_ₙ represented the residual error.

Model assumptions were validated using a simulation-based approach to assess residual uniformity, detect outliers, and evaluate zero inflation, confirming an adequate fit to the data [22]. Statistical significance of fixed effects was assessed using 95% CI and p-values (set at *p* < 0.05) obtained from the model output. For the categorical predictor load, which included three levels (45%, 65%, 85% 1RM), post-hoc pairwise comparisons of estimated marginal means were performed using Tukey’s adjustment to control for multiple testing. In addition to the model results, we present descriptive statistics for the different outcomes using means, standard deviations (SD), and ranges.

Statistical analysis was performed using the R statistical computing environment (R Core Team, Vienna, Austria, version 4.5.0, 2025) via the RStudio integrated development environment for R (Posit Software, PBC, Boston, MA, version 2025.05.0.496). Graphs were made using the “ggplot2” R package (version 3.5.2; Wickham 2016). Mixed-effect regression models were fitted using the “lme4” R package (version 1.1–37; Bates, Martin, Bolker, and Walker 2015). Estimated marginal means were calculated using the “emmeans” R package (version 1.11.1, Russell 2025).

## Results

*Gaze Strategy*: Participants used the bar strategy in 747 out of 960 observations (mean proportion = 77.81, 95% CI [0.75, 1.00]).

*Accuracy*: Descriptive statistics (mean, SD, and range) of absolute error are presented in Table 1. The average absolute error was 1.5 repetitions (SD = 2.3, range: 0–15) across all lifted loads, VL thresholds, and gaze strategies.

**Table 1:** Descriptive statistics of average absolute error across loads, VL thresholds, and gaze strategies

Characteristic	Mean (SD); Range
Overall	1.5 (2.3); 0.0–15.0
Load 45%	1.9 (3.2); 0.0–15.0
Load 65%	1.6 (2.0); 0.0–10.0
Load 85%	0.9 (1.2); 0.0–7.0
Threshold 20%	1.9 (2.4); 0.0–15.0
Threshold 40%	1.1 (2.1); 0.0–13.0
Strategy No-Bar	1.9 (2.6); 0.0–13.0
Strategy Bar	1.4 (2.2); 0.0–15.0

*SD* Standard deviation

To aid interpretation, we present exponentiated estimates from the negative binomial generalized linear mixed-effects model to obtain rate ratios, which were then transformed to indicate changes in the expected number of repetitions relative to the reference condition (Table 2). The model’s intercept was estimated at 3.33 repetitions (95% CI [2.00, 5.57]), representing the average absolute error in detecting a 20% VL threshold during sets loaded at 45% 1RM using a no-bar gaze strategy. Relative to this reference condition, we found statistically significant effects indicating greater accuracy (lower error) for 40% VL, 85% 1RM, and bar strategy, but not for 65% 1RM or mental fatigue (Table 2). Post-hoc pairwise comparisons of estimated marginal means for load, adjusted using Tukey’s method, indicated that error was significantly lower at 85% 1RM compared with both 45% 1RM (*p* < 0.0001) and 65% 1RM (*p* < 0.0001), whereas the difference between 45% and 65% 1RM was not statistically significant (*p* = 0.305).

**Table 2:** Outputs of the negative binomial general linear mixed-effects model for the Coach’s Eye accuracy

Parameter	Error (n. repetitions)	95% CI	*P*-value
Fixed Effects			
(Intercept)	3.33	(2.00, 5.57)	< 0.001*
Threshold (40%)	−1.60	(−1.88, −1.26)	< 0.001*
Load (65%)	−0.48	(−1.01, 0.18)	0.142
Load (85%)	−1.59	(−1.93, −1.16)	< 0.001*
Strategy (Bar)	−1.23	(−1.99, −0.03)	0.045*
Fatigue (0–100)	−0.01	(−0.03, 0.01)	0.446
Random Effects			
σ^2^	0.99		
τ_00 participant_	0.32		
ICC	0.24		
N	20		
Observations	960		
R^2^ Marginal/Conditional	0.146/0.355		

^*^Significance < 0.05

*CI* confidence interval, *ICC* intraclass correlation coefficient

Intercept: 20% VL, 45% 1RM, no-bar strategy

σ^2^: within-subject variability

Model estimates were exponentiated and transformed to reflect changes in the expected number of repetitions compared to the reference condition

*Fatigue*: The average perceived mental fatigue reported by participants was 33.6 (SD = 20.9; range: 0 to 85).

*Questions*: In response to Question 1 (“How accurate do you think you were in detecting the velocity loss thresholds?”), participants reported an average perceived accuracy of 54.4 (SD = 18.18, range: 15 to 90). In response to Question 2 (“Did you implement the new strategy? If so, how effective were you in implementing it?”), all participants indicated they had implemented the bar strategy, with an average perceived effectiveness score of 80.75 (SD = 14.8; range: 40 to 100).

## Discussion

In this study, we investigated the trainability of the bar gaze strategy for tracking barbell VL and whether its use was associated with greater accuracy in detecting repetitions corresponding to 20% and 40% VL thresholds during RT exercises. Following the instructional video, most participants adopted the strategy, and overall accuracy was 1.5 repetitions on average across all conditions. Greater accuracy was found when participants used the bar strategy, attempted to detect higher VL thresholds, and observed exercises performed with heavier loads, whereas perceived mental fatigue had a negligible effect.

In our previous study, participants spontaneously used the bar strategy in approximately 48% of observations, and the average absolute error across all conditions (thresholds, loads, views) was 2.6 repetitions [19]. Use of the bar strategy was associated with a 1.7-repetition decrease in error. The error also decreased when detecting higher VL thresholds and with heavier loads – a finding repeated in the current study but not discussed further here, as it was addressed previously [19]. Building upon these findings, we hypothesized that with explicit instruction, the use of the bar strategy would increase and subsequently be associated with greater accuracy. Our findings support this hypothesis: after watching an instructional video, the use of the bar strategy was ~ 78%, and the average absolute error across all conditions was 1.5 repetitions, with an additional 1.23-repetition reduction when the bar strategy was effectively implemented.

Interestingly, in the current study, participants who used a no-bar strategy showed an average absolute error of 1.9 repetitions compared to 2.9 in the previous study [19]. Since the same cohort participated in both studies, an intuitive explanation for this finding would be a learning effect. However, the inclusion of a washout period of at least 8 weeks, with most participants having a 4–6-month washout, reduces the likelihood of such an effect. An alternative plausible explanation is the perceptual calibration of perceived VL participants were exposed to during the instructional video, showing the gradual decrease in bar velocity with labels for 20% and 40% VL. This may have helped all participants refine their internal reference for judging VL, independent of gaze behavior. Notably, a perceptual calibration was not provided in our previous study. Alternatively, the improvement may have resulted from the explicit instructions participants received, which could have increased task engagement and reduced attention to irrelevant cues (e.g., the trainee’s facial expression, static body parts, or areas distant from the moving bar), even when the bar strategy was not fully adopted. Still, when participants used the bar strategy, accuracy was highest overall, highlighting its potential superiority as a perceptual aid. That said, to properly disentangle potential learning effects from the impact of gaze training, a randomized controlled trial design is needed, including a control group that repeats the task without receiving gaze training.

To our knowledge, this is the first study to investigate the effectiveness of training a gaze strategy for tracking barbell VL in RT, and more broadly, for tracking an object’s velocity changes in general. In sports science, gaze training has primarily focused on two domains: anticipation skills (e.g., predicting an opponent’s action) and aiming skills (e.g., putting in golf) [23], with effectiveness demonstrated across various sports, such as soccer [24, 25], volleyball [26], basketball [27], handball [28], and golf [29, 30]. While specific gaze training protocols varied by sport, the methodological approach was consistent and aligned with ours: identifying an effective gaze strategy, demonstrating it, providing feedback, and testing implementation [31]. For example, high-level basketball players show more prolonged final fixation on the hoop before free throws than lower-level players [32]. Studies that train lower-level players to use this strategy typically first introduce the strategy’s key characteristics, then provide participants with feedback comparing their gaze patterns to those of high-level players, and ultimately retest accuracy to assess improvement [27, 33, 34]. Our study extends this research line by showing, for the first time, that gaze strategies can be trained to track changes in object velocity. However, like previous studies, our single-experiment design offers limited insight into the long-term retention of the learned strategy. Future studies should examine whether the trained gaze strategy is retained over time and whether periodic calibration or refresher sessions are necessary to maintain accuracy.

Another important finding of our study was the relatively high levels of mental fatigue reported by participants, with an average score of 33.6 on a 0–100 point scale. This is substantially higher compared to the participants’ average mental fatigue reported in our previous study, which was only 22.4 [19]. Mental fatigue typically arises from prolonged cognitively demanding tasks [35] and has been shown to impair performance in certain contexts [36]. However, in our study, mental fatigue was not associated with decreased accuracy, aligning with other evidence suggesting that it does not consistently impair performance [37]. The higher mental fatigue reported by participants in the current study can be explained by the greater cognitive load imposed by gaze training. In the current study, the intervention required participants to memorize anchor points (first repetition in a set), imagine and retain the reference lines representing the lower and upper extremes of the object’s range of movement, and sustain focused visual tracking of the barbell, all of which are known to increase visual attention and working memory demands [37, 38]. In contrast, in the previous study, participants likely relied more on spontaneous processing during the task, requiring less deliberate cognitive control. Interestingly, an exploratory inspection of the data suggested that mental fatigue levels were similar between participants who used the bar strategy and those who did not. Therefore, similar to the improved accuracy across gaze strategies, the elevated mental fatigue may stem from the explicit instructions alone, encouraging greater engagement with the task. Notably, while watching the 9-minute instructional video may have contributed to the higher mental fatigue reported in the second experiment, we did not measure baseline fatigue levels at the beginning of the session, so its specific effect cannot be determined. Future studies should examine whether the associated mental fatigue diminishes as the strategy becomes more familiar and automatic.

While our study focused on RT coaches, others have examined trainees’ perception of VL as a tech-free VBT alternative. Since coaches can choose to rely on either their own judgment or their trainee’s, comparing their respective accuracy is valuable. Previous studies have reported that trainees perceive VL thresholds with an average absolute error range of 1–2 repetitions [16, 17]. For example, Dello Iacono et al. reported that trainees perceived VL with an average absolute error of 1 repetition during the barbell bench press but found reduced accuracy at heavier loads and no significant effect of VL threshold on accuracy [17]. In contrast, here we observed improved accuracy at heavier loads, with a mean absolute error of 1.9, 1.6, and 0.9 repetitions at 45%, 65%, and 85% 1RM, respectively, and greater accuracy at the 40% VL threshold. Our findings align with a recent study by Dello Stritto et al., which reported that trainees’ accuracy improved at heavier loads and at greater VL thresholds (i.e., 40% vs. 20%) [18]. However, their protocol included a four-session familiarization period to calibrate participants’ perception of VL, a step not included in our study nor in Dello Iacono et al. [17]. Apart from familiarization, the discrepancies between coaches’ and trainees’ perceptions of VL likely stem from the distinct perceptual cues available to them. While trainees likely use a combination of non-visual cues, such as exertion, fatigue, and proprioception, when perceiving their own VL, coaches can use solely visual cues. To clarify these discrepancies and guide the practical application of tech-free VBT, further studies should directly compare the accuracy of coaches and trainees across varying conditions (e.g., familiarization, load, VL threshold) to determine who is more accurate and under what conditions.

This study has several limitations. First, we developed the gaze strategy categories (bar, no-bar) based on findings from our previous study with a small sample (*n* = 20). However, other effective strategies may emerge with larger cohorts, for example, fixating on the top of the barbell’s range of motion and waiting for it to reach that point. Second, our design establishes an association, but not causation, between the bar strategy and greater accuracy. A randomized trial, including a control group that repeats the task without instructions, would be needed to draw causal inferences. Third, pausing the video to present the mental fatigue scale after each VL response may have disrupted participants’ ability to compare later repetitions to the first in the set, which served as the reference (i.e., 100%). While this methodological choice allowed us to assess mental fatigue at each VL threshold, it potentially increased error and impaired external validity, as in real-world training, once the VL threshold is reached, the set is terminated. Fourth, our method for calculating absolute error may underestimate true error when the VL threshold is not exceeded. If a participant misjudged the threshold near the end of a set, the small remaining repetition count might artificially lower the perceived error, even if the trainee could have continued. While this calculation does not account for the number of additional repetitions that could have been completed before exceeding the VL threshold, the extent of this error remains unknown, as trainees voluntarily terminated the sets. Finally, the controlled lab-based video task included only two barbell exercises and lacked real-world gym distractions, limiting the generalizability of our findings. To enhance external validity, future studies should assess coaches’ accuracy in live gym settings and across a broader range of exercises, including those performed with free weights and machines.

## Conclusion

This study is the first to demonstrate the trainability of a gaze strategy for perceiving barbell VL and the second to examine RT coaches’ accuracy in detecting repetition VL thresholds. We found that a short instructional video enabled coaches to learn and apply the bar strategy and that its use was associated with improved accuracy. These findings suggest that RT coaches’ observational judgment – the Coach’s Eye – can serve as a complementary tool for implementing VBT, reducing dependence on velocity-tracking devices, and increasing accessibility to the VBT method.

## Supplementary Information

Supplementary Material 1

## Data Availability

The dataset used in the current study and the R statistical analysis code are available online at: https://osf.io/yt6wa.

## Abbreviations

RT: Resistance training
VBT: Velocity–based training
VL: Velocity loss
1RM: One repetition maximum
SD: Standard deviation
CI: Confidence interval

## References

[1] Weakley J, Mann B, Banyard H, McLaren S, Scott T, Garcia-Ramos A. Velocity-based training: from theory to application. Strength Cond J. 2021;43:31.

[2] Pareja-Blanco F, Rodríguez-Rosell D, Sánchez-Medina L, et al. Effects of velocity loss during resistance training on athletic performance, strength gains and muscle adaptations. Scand J Med Sci Sports. 2017;27:724–35. 27038416 10.1111/sms.12678

[3] Alcazar J, Cornejo-Daza PJ, Sánchez-Valdepeñas J, Alegre LM, Pareja-Blanco F. Dose–response relationship between velocity loss during resistance training and changes in the squat force–velocity relationship. Int J Sports Physiol Perform. 2021;16(12):1736–45. 34044366 10.1123/ijspp.2020-0692

[4] Sánchez-Medina L, González-Badillo JJ. Velocity loss as an indicator of neuromuscular fatigue during resistance training. Med Sci Sports Exerc. 2011;43:1725–34. 21311352 10.1249/MSS.0b013e318213f880

[5] Cowley N, Nicholson V, Timmins R, Munteanu G, Wood T, García-Ramos A, et al. The effects of percentage-based, rating of perceived exertion, repetitions in reserve, and velocity-based training on performance and fatigue responses. J Strength Cond Res. 2025;39:e516. 39787033 10.1519/JSC.0000000000005026

[6] Dorrell HF, Smith MF, Gee TI. Comparison of velocity-based and traditional percentage-based loading methods on maximal strength and power adaptations. J Strength Cond Res. 2020;34:46. 30946276 10.1519/JSC.0000000000003089

[7] Held S, Hecksteden A, Meyer T, Donath L. Improved strength and recovery after velocity-based training: a randomized controlled trial. Int J Sports Physiol Perform. 2021;16(8):1185–93. 33547265 10.1123/ijspp.2020-0451

[8] Montalvo-Pérez A, Alejo LB, Valenzuela PL, Gil-Cabrera J, Talavera E, Lucia A, et al. Traditional versus velocity-based resistance training in competitive female cyclists: a randomized controlled trial. Front Physiol. 2021;12:586113. 33716761 10.3389/fphys.2021.586113 PMC7947617

[9] Thompson SW, Olusoga P, Rogerson D, Ruddock A, Barnes A. Is it a slow day or a go day? The perceptions and applications of velocity-based training within elite strength and conditioning. Int J Sports Sci Coach. 2023;18:1217–28.

[10] Bautista IJ, Chirosa IJ, Chirosa LJ, Martín I, González A, Robertson RJ. Development and validity of a scale of perception of velocity in resistance exercise. J Sports Sci Med. 2014;13:542–9. 25177180 PMC4126290

[11] Bautista IJ, Chirosa IJ, Robinson JE, Chirosa LJ, Martínez I. Concurrent validity of a velocity perception scale to monitor back squat exercise intensity in young skiers. J Strength Cond Res. 2016;30:421. 26244826 10.1519/JSC.0000000000001112

[12] Romagnoli R, Civitella S, Minganti C, Piacentini MF. Concurrent and predictive validity of an exercise-specific scale for the perception of velocity in the back squat. Int J Environ Res Public Health. 2022;19:11440. 36141713 10.3390/ijerph191811440 PMC9517416

[13] Romagnoli R, Piacentini MF. Perception of velocity during free-weight exercises: difference between back squat and bench press. J Funct Morphol Kinesiol. 2022;7:34. 35466269 10.3390/jfmk7020034 PMC9036296

[14] Sindiani M, Lazarus A, Iacono AD, Halperin I. Perception of changes in bar velocity in resistance training: accuracy levels within and between exercises. Physiol Behav. 2020;224:113025. 32585167 10.1016/j.physbeh.2020.113025

[15] da Silva DG, da Silva RFB, Gantois P, Nascimento VB, Nakamura FY, Fonseca F. Accuracy and reliability of perception of bar velocity loss for autoregulation in resistance exercise. Int J Sports Sci Coach. 2024;19:1622–31.

[16] Shaw M, Thompson S, Myranuet PA, Tonheim H, Nielsen J, Steele J. Perception of barbell velocity: can individuals accurately perceive changes in velocity? Int J Strength Cond. 2023;3(1).

[17] Dello Iacono A, Watson K, Marinkovic M, Halperin I. Perception of bar velocity loss in resistance exercises: accuracy across loads and velocity loss thresholds in the bench press. Int J Sports Physiol Perform. 2023;18:488–94. 36928000 10.1123/ijspp.2022-0298

[18] Dello Stritto E, Gramazio A, Romagnoli R, Piacentini MF. Temporal stability and practical relevance of velocity and velocity-loss perception in back squat. Appl Sci. 2025;15:7252.10.3390/jfmk1103034542783639

[19] Dello Iacono A, Henry S, Ben-Ari A, Halperin I, Carey L. The coach’s eye: a randomized repeated-measure observational study assessing coaches’ perception of velocity loss during resistance training exercises. Sports Med Open. 2025;11:83. 40634547 10.1186/s40798-025-00890-1 PMC12240891

[20] Kumle L, Võ M-H, Draschkow D. Estimating power in (generalized) linear mixed models: an open introduction and tutorial in R. Behav Res Methods. 2021;53:2528–43. 33954914 10.3758/s13428-021-01546-0 PMC8613146

[21] Lee KA, Hicks G, Nino-Murcia G. Validity and reliability of a scale to assess fatigue. Psychiatry Res. 1991;36:291–8. 2062970 10.1016/0165-1781(91)90027-m

[22] Florian H. DHARMa: Residual diagnostics for hierarchical (multi-level / mixed) regression models. CRAN Contrib Packag. 2016.

[23] Ziv G, Lidor R. Gaze and visual perception in sport. Milton Park: Taylor & Francis; 2025.

[24] Zhao J, Gu Q, Zhao S, Mao J. Effects of video-based training on anticipation and decision-making in football players: a systematic review. Front Hum Neurosci. 2022;16:945067. 36438631 10.3389/fnhum.2022.945067 PMC9686440

[25] Wood G, Wilson MR. Quiet-eye training for soccer penalty kicks. Cogn Process. 2011;12:257–66. 21318734 10.1007/s10339-011-0393-0

[26] Zali F, Arabameri E, Shahbazi M. The effect of quiet eye training on the gaze behavior and performance of players in volleyball service. Int J Mot Control Learn. 2023;5:29–34.

[27] Harle SK, Vickers JN. Training quiet eye improves accuracy in the basketball free throw. Sport Psychol. 2001;15(3):289–305.

[28] Abernethy B, Schorer J, Jackson RC, Hagemann N. Perceptual training methods compared: the relative efficacy of different approaches to enhancing sport-specific anticipation. J Exp Psychol Appl. 2012;18:143–53. 22564086 10.1037/a0028452

[29] Vine SJ, Moore L, Wilson MR. Quiet eye training facilitates competitive putting performance in elite golfers. Front Psychol. 2011;2:8. 21713182 10.3389/fpsyg.2011.00008 PMC3111367

[30] Vine SJ, Wilson MR. Quiet eye training: effects on learning and performance under pressure. J Appl Sport Psychol. 2010;22:361–76.

[31] Vickers JN. Origins and current issues in quiet eye research. Curr Issues Sport Sci. 2016;1:1–1.

[32] Vickers JN. Control of visual attention during the basketball free throw. Am J Sports Med. 1996;24:S93-7. 8947439

[33] Rienhoff R, Fischer L, Strauss B, Baker J, Schorer J. Focus of attention influences quiet-eye behavior: an exploratory investigation of different skill levels in female basketball players. Sport Exerc Perform Psychol. 2015;4:62–74.

[34] Vickers JN, Causer J, Vanhooren D. The role of quiet eye timing and location in the basketball three-point shot: a new research paradigm. Front Psychol. 2019;30(10):2424. 10.3389/fpsyg.2019.02424 PMC6836760 31736825

[35] Craik FIM. Effects of distraction on memory and cognition: a commentary. Front Psychol. 2014;5:841. 25120527 10.3389/fpsyg.2014.00841 PMC4114291

[36] Dallaway N, Lucas SJE, Ring C. Cognitive tasks elicit mental fatigue and impair subsequent physical task endurance: effects of task duration and type. Psychophysiology. 2022;59:e14126. 35726493 10.1111/psyp.14126 PMC9786280

[37] Pessiglione M, Blain B, Wiehler A, Naik S. Origins and consequences of cognitive fatigue. Trends Cogn Sci. 2025. 10.1016/j.tics.2025.02.005. 40169294 10.1016/j.tics.2025.02.005

[38] Liu P, Forte J, Sewell D, Carter O. Cognitive load effects on early visual perceptual processing. Atten Percept Psychophys. 2018;80:929–50. 29363029 10.3758/s13414-017-1464-9

