# Supplementary material for: Sharpening the Coach’s Eye: An Observational Study Investigating the Trainability of An Eye-Tracking Strategy for Perceiving Barbell Velocity Loss in Resistance Training
Source: Sports Med Open. 2026 Mar 11;12:28. doi: 10.1186/s40798-026-01003-2 (PMC12979748; doi:10.1186/s40798-026-01003-2)
Supplement: Supplementary file 1 — Supplementary Material 1 [file 40798_2026_1003_MOESM1_ESM.docx]

**Supplementary File 1**

**Article title:** *Sharpening the Coach’s Eye: An Observational Study Investigating the Trainability of an Eye-Tracking Strategy for Perceiving Barbell Velocity Loss in Resistance Training*

**Journal:** *Sports Medicine – Open*

**Authors:** Asaf Ben-Ari^1,2,3^, Scott Henry^1^, Israel Halperin^2,3^, Laura Carey^1†^, Antonio Dello Iacono^1†^.

**Authors’ information:**

^1^Sport and Physical Activity Research Institute (SPARI), Division of Sport, Exercise and Health, School of Health and Life Sciences, University of the West of Scotland, United Kingdom.

^2^Department of Health Promotion, School of Public Health, Faculty of Medical and Health Sciences, Tel Aviv University, Israel.

^3^Sylvan Adams Sports Institute, Tel Aviv University, Israel.

^†^Equal senior authors.

**Corresponding author:** Antonio Dello Iacono, Phone: +44 7445454334, Email: antonio.delloiacono@uws.ac.uk

**Video Recordings Procedure**

All video recordings were captured at an acquisition rate of 25 frames per second, using two full high-definition (Video Resolution: 1920x1080 pixels) digital video cameras (Casio Exilim 100, Casio, Japan) mounted on tripods 1.2 meters from the ground, and positioned in the frontal (front view) and sagittal (side view at a 90-degree angle) planes, at distances of 3 meters and 2 meters, respectively. These angles and distances were chosen to: 1) replicate the typical observational positions used by resistance training (RT) coaches in practice, and 2) provide relevant information for analyzing the participants' gaze strategies during the experimental task.

**Trainees**

The video recordings featured two trainees (one female, one male), both sports and science students at the University of the West of Scotland. Each provided written informed consent to be recorded and for their recordings to be used in this study. Both trainees had a minimum of five years of RT experience and trained regularly, at least three times per week.

**One-repetition Maximum (1RM) Test**

Each trainee participated in a single laboratory session. The session began with a 1RM assessment for the barbell back squat and barbell bench press. To prepare, trainees first completed an 8-minute general warm-up: 5 minutes of individualized, self-selected activity on either a cycle ergometer or an arm ergometer, followed by 3 minutes of dynamic stretching that targeted both upper and lower body muscles. This was followed by an exercise-specific warm-up, consisting of 2–4 sets of 2–3 repetitions with progressively heavier loads (concentric velocity range: 1.4–0.3 m·s⁻¹). The 1RM was then estimated for each exercise using the protocol outlined by Loturco et al. [1].

**Exercises**

After a 10-minute rest period, trainees performed three sets of each exercise (back squat and bench press) to self-selected task failure, defined as the point at which they felt unable to complete another repetition. Each exercise was performed with three loads: 85%, 65%, and 45% of 1RM, in descending order, that is, starting with the heaviest load and progressing to the lightest (i.e., 85%-65%-45% 1RM). Trainees were instructed to execute the concentric phase of each repetition as fast as possible, while maintaining a controlled eccentric phase lasting approximately 2 seconds, until they touched a bench (barbell back squat; adjusted to ensure a knee angle of 80-90 degrees) or the bar touched their chest (barbell bench press). An 8-minute rest period was provided between sets and exercises. Concentric barbell velocity was recorded for every repetition using a linear positioning transducer (Chronojump Boscosystem, Barcelona, Spain) attached to the barbell with a string tether positioned perpendicular to the ground. The validity and reliability of the linear positioning transducer were confirmed by others [2].

All exercises were performed with a free 20-kg barbell (Origin, Edinburgh, United Kingdom) and calibrated weight plates (Eleiko, Halmstad, Sweden). No lifting aids (e.g., weightlifting shoes, belts, knee wraps, or straps) were used. To minimize the impact of fatigue or external influences, trainees were instructed to avoid intense RT involving the same exercises in the 24 hours prior to testing, and to refrain from consuming heavy meals, caffeine, or ergogenic supplements for at least 2 hours before the session.

**Scales**

**Mental Fatigue Scale**

During the experimental session, participants rated their perceived mental fatigue using the visual analog scale presented below (Figure S1). The researcher asked participants to rate their mental fatigue (“How mentally fatigued are you?”) from 0 (“no fatigue”) to 100 (“maximal fatigue—I can’t carry on the task”) [3]. The following electronic version of the scale was projected on the screen immediately after each answer and/or at the end of a video if the participants provided no answer regarding the perception of VL.

**Figure S1:** Mental fatigue scale.


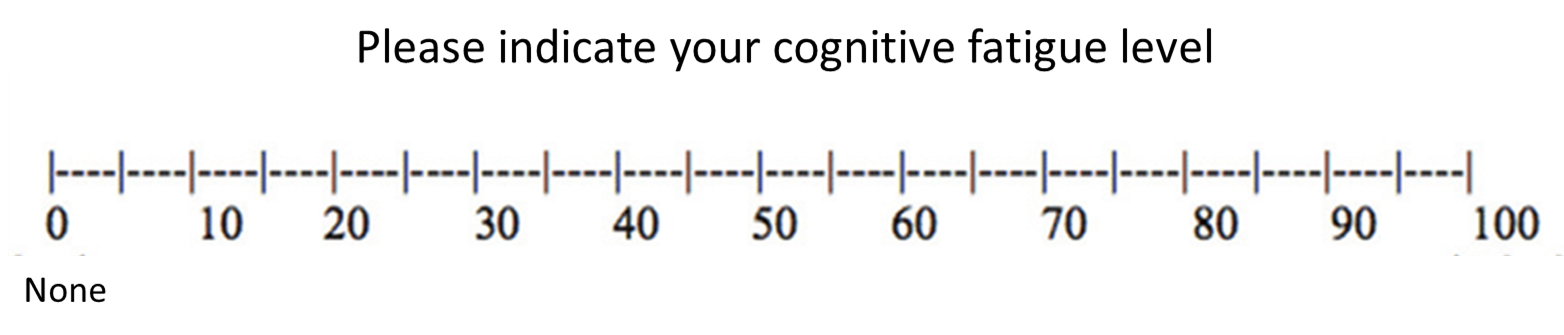


**References:**

1. Loturco I, Iacono AD, Nakamura FY, Freitas TT, Boullosa D, Valenzuela PL, Pereira LA, McGuigan MR (2021) The Optimum Power Load: A Simple and Powerful Tool for Testing and Training. Int J Sports Physiol Perform 17(2):151-9.

2. Vivancos AL, Zambudio AC, Ramírez FC, Águila AD, Castrillón FJO, Pardo PJM (2014) OC14 Reliability and validity of a linear position transducer for strength assessment. Br J Sports Med 48:A5–A5.

3. Lee KA, Hicks G, Nino-Murcia G (1991) Validity and reliability of a scale to assess fatigue. Psychiatry Res 36:291–298.
